# INEQUALITY IN AWARENESS, TREATMENT, AND CONTROL OF VISUAL IMPAIRMENTS AMONG OLDER ADULTS AND ELDERLY IN INDIA

**DOI:** 10.1093/geroni/igad104.3079

**Published:** 2023-12-21

**Authors:** Rajeev Singh

**Affiliations:** International Institute for Population Sciences, Mumbai, Mumbai, Maharashtra, India

## Abstract

Globally, 1.1 billion people have some form of visual impairment, and 90 percent of them live in low and middle-income countries. The prevalence of visual impairment is increasing, and the likelihood of increasing this trend is also higher. Yet, there is little evidence on the extent of awareness, treatment, and control (ATC) of visual impairment in India. We estimate the prevalence and ATC of visual impairment among older adults and the elderly across socioeconomic groups. We used the unit data from the first round of the Longitudinal ageing study of India (LASI), 2017-18, bivariate analysis, estimated age sex-adjusted prevalence, logistic regression, concentration index, and concentration curve used in the analyses. Among the 45+ population, 30.18% have any form of visual impairment, around 47.96% have awareness about the visual impairment, 17.21% have taken treatment for vision-related issues, and 12.70% have control vision after treatment. The prevalence was higher among the illiterate poor and females; however, the awareness, treatment, and control were higher among those with higher education who belongs to the richer richest wealth quintile. The concentration index and concentration curve suggest that higher prevalence was concentrated among the poor, and contrary to that, awareness treatment and control were concentrated among richer. To deal with it, public health services from the primary, secondary, and tertiary levels may be integrated for effective care of growing, underdiagnosed, and untreated visual impairments among older adults and the elderly in India.

